# Escalation of radiotherapy dose in large locally advanced drug-resistant gastrointestinal stromal tumors by multi-shell simultaneous integrated boost intensity-modulated technique: a feasibility study

**DOI:** 10.1186/s13014-022-02179-z

**Published:** 2022-12-28

**Authors:** Haixia Cui, Ying Li, Wei Huang, Wenli Lu, Xin Yi

**Affiliations:** Department of Oncology, The First Affiliated Hospital of Chongqing Medical University, Chongqing, 400016 China

**Keywords:** GISTs, Locally advanced drug-resistant, Large tumor, Multi-shells SIB-IMRT, EUD

## Abstract

**Background:**

Resistance to conventional dose schemes and radiotoxicity of healthy tissue is a clinical challenge in the radiation therapy of large locally advanced drug-resistant gastrointestinal stromal tumor (LADR-GIST). This study aimed to assess the feasibility of using multi-shell Simultaneous Integrated Boost Intensity-Modulated modality (SIB-IMRT) strategy to provide a safe and effective escalation dose regimen for LADR-GIST.

**Methods:**

7 patients with LADR-GIST were selected in this study. The modified SIB-IMRT plans for all patients were generated by delivering different escalation-dose gradients to four ring shaped regions (shells) within the gross tumor volume (GTV). The doses of the central volume of the tumor (GTV_center_) were escalated up to 70–92.5 Gy (25 fractions), while the doses of planning target volume (PTV) and shell-1 were kept at 50.0 Gy. Based on different escalation-dose gradients, the modified SIB-IMRT plans were divided into four groups (SIB-IMRT groups). For comparison purposes, plans obtained by conventional IMRT technique (Con-IMRT) with 50 Gy (25 fractions) were also generated for all patients (Con-IMRT group). All plans were normalized to cover 95% of the PTV with the prescribed dose of 50.0 Gy. The equivalent uniform dose (*EUD*), relative equivalent uniform dose (*rEUD*), dose volume histogram (DVH), dose profile, conformity index (CI) and monitor unit (MU) were evaluated in five groups. The Friedman Test was performed to determine whether there were significant differences (*P* < 0.05).

**Results:**

Compared with the Con-IMRT group, the *EUD* of GTV *(EUD*_*GTV*_) and *rEUD* of SIB-IMRT groups were improved when escalation-dose gradient was increased, and the improvement became significant when the escalation-dose gradient reached 20% of the prescription dose. The *rEUD* tended to be stable as the escalation-dose gradient went up to 25% of the prescription dose. There were no significant differences in CIs and DVH metrics for OARs between the Con-IMRT group and any SIB-IMRT group, but the significant differences were observed between the SIB_10_-IMRT group and the SIB_25_-IMRT group. For the SIB-IMRT groups, as the dose gradient became steeper in the dose profiles, the higher dose was mainly accumulated in the inner part of GTV accompanied with a higher MU.

**Conclusions:**

The proposed multi-shell SIB-IMRT strategy is feasible in dosimetry for LADR-GIST and can acquire higher therapeutic gain without sacrifice of healthy tissues. It appears that the scheme of delivering 20% of the prescribed escalation-dose gradient to the target volume can provide satisfactory dose irradiation for LADR-GIST, and it should be evaluated in future clinical study.

## Background

Gastrointestinal stromal tumors (GISTs) are uncommon neoplasms arising from the mesenchymal tissue of the gastrointestinal tracts [1]. Tumor size, mitosis rate, tumor location, kinase mutation status, and tumor rupture have been widely studied and considered as prognostic indicators [2]. The mutations in tyrosine kinase receptor (KIT) and/or platelet-derived growth factor receptor alpha (PDGFRA) gene have been considered as main factors of the pathogenesis of GISTs. Resection and/or tyrosine kinase inhibitors (TKIs) therapy is always the first choice for GISTs treatment [3]. Although TKIs have greatly improved the therapeutic effect of advanced GISTs, the secondary drug resistance is still common [1]. Some tumors, especially locally advanced drug-resistant gastrointestinal stromal tumor (LADR-GIST), are often technically unresectable and easily resistant to TKIs therapy. As for patients with technically unresectable tumors, there are only few treatment options left once they are resistant to systemic TKIs therapy. Therefore, the management of LADR-GIST has emerged as a challenging clinical problem. Under such circumstances, radiotherapy maybe a valuable option for LADR-GIST management [4].

Since GISTs is traditionally considered to be insensitive to radiation, radiotherapy is not recommended as a therapeutic option and is only for palliative intent in the current treatment guidelines [5, 6]. There was not a large-scale, randomized, prospective trial to evaluate the efficacy of radiotherapy for GISTs. So far, a few case reports showed that GISTs were not uniformly radioresistant and that radiotherapy could be a valuable alternative in GISTs management [7, 8]. Due to missing consensus on radiotherapy for patients with GISTs, various attempts of radiation dose schemes have been reported [4, 9]. In a retrospective study of 15 patients, Cuaron et al. [9] suggested that GISTs were more sensitive to a higher radiation dose. In our previous study [10], the prescribed dose of PTV was set to 50.4 Gy in 28 fractions, while the dose for the center of GTV was boosted to 62–64 Gy. Based on Choi criteria [11], the partial response of the three LADR-GIST patients showed that a reasonable boosted dose for the center of tumor may obtain an effective tumor control with negligible treatment toxicities. These reports demonstrated that a higher and heterogeneous dose distribution in radiotherapy may offer a valuable alternative option for management of LADR-GISTs.

Actually, a uniform dose distribution is commonly applied within target volume and the maximum dose is limited within 110%–115% of the prescription in Con-IMRT. However, the prescription of a homogeneous dose cannot meet the hypoxia requirement of large tumors, as the central area of large tumors has obvious hypoxic changes and should be treated with higher radiation doses [12, 13]. To improve the tumor response in Con-IMRT, we generally increase the radiation dosage for the whole target volume. Nevertheless, a higher radiation dose delivered to bulky tumors in Con-IMRT could cause serious side effects as a result of a significant increased irradiation of healthy tissue. Compared with Con-IMRT and sequential IMRT technique, SIB-IMRT allow a dose boost to different target volumes of the tumor and increase tumor response without significant increase of healthy tissue irradiation [14, 15]. Due to the particularity of the LADR-GIST, it is a challenge to deliver a stereotactic-like dose to tumor using SIB-IMRT modality. Fortunately, the shell-structure optimization is able to provide a better dose conformity and control dose gradients around the target volume without compromise of target volume coverage [16, 17]. Therefore, the shell-structure optimization has been widely used in Stereotactic body radiotherapy (SBRT) plans, and has helped the SBRT plan to get a better conformity, lower radiation dose for OARs, and smaller low-dose areas of normal tissue [18, 19]. Given the above, the shell-structures applied in SIB-IMRT may have the potential to improve the tumor response without significant increase in the radiotoxicity of the adjacent normal in LADR-GISTs. In this paper, taking the advantages of multi-shell optimizing and SIB-IMRT technique, we planned a dose-escalation study for large LADR-GIST. This new planning scheme was named as multi-shell SIB-IMRT. The focus of this study was to explore a safe and effective radiation regimen for the LADR-GIST, which can generate a non-uniform and higher dose distribution inside the GTV without exposing OARs to higher radiation doses.

## Methods and materials

### Patient and volume definition

**Fig. 1:**
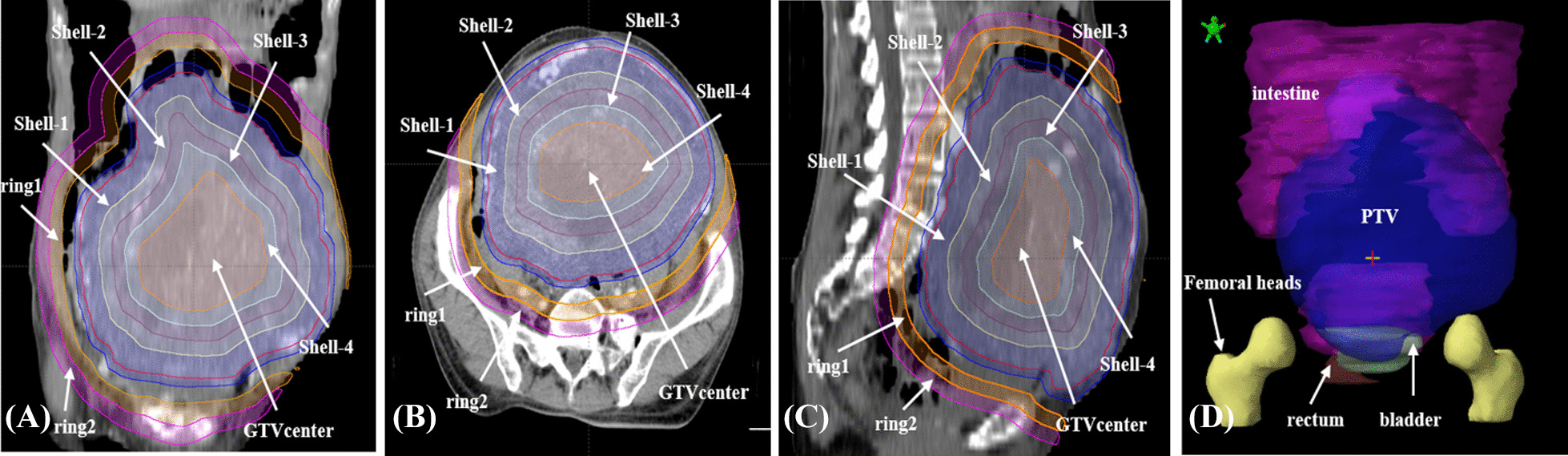
Axial (**A**), sagittal (**B**), and coronal (**C**) view of typical ring regions and 3D display of OARs and PTV (**D**) in patient 1

For dosimetry analysis, 7 patients with LADR-GIST treated in our center from January 2016 to June 2022 were selected in this study, and their tumors’ maximum diameters in diagnostic CT imaging were all over 10 cm. The median age of patients was 58 years. These patients underwent R_0_ resection of the primary tumor followed by systemic TKIs therapies, and they had never been treated with radiotherapy or embolization before. A few years later (1–10 years), tumor recurrence and/or metastasis, as well as drug resistance were found. Moreover, lesion progression and bulky tumor were detected in these patients (details in Table 1). The GTV diameter ranged from 12.0 to 20.0 cm (17.7 cm ± 2.6 cm). The total abdomen pelvic region was covered in the planning CT scan (GE Medical Systems, Milwaukee, WI). Target volume and organs at risk (OARs) were contoured with Eclipse™ treatment planning system version 13.5 (Varian Medical Systems, Palo Alto, CA) by the same attending oncologist, and were reviewed by a senior oncologist. We registered the relevant MRI sequences and/or contrasted CT images with the treatment planning CT before contouring. The GTV of the LADR-GIST was delineated on the registration images. After that, the PTV was obtained by GTV plus 5 mm margin. The clinical target volume (CTV) was not defined considering the low rate of lymph node metastasis (1–2%) in GISTs [1, 3]. The OARs mainly included rectum, bladder, intestine, and femoral heads [10]. The normal tissue (NT) structure was defined as the body minus PTV. Ring 1 was defined as 2 cm of PTV margin minus 1 cm of PTV margin, and ring 2 was defined as 3 cm of PTV margin minus 2 cm of PTV margin. In addition, GTV was divided into 5 parts by 4 shells based on the concentric contraction within GTV. The first inner shell (shell-1) was 1–1.5 cm concentric contraction from the outer contour of GTV. Each subsequent inner shell (shell-2, shell-3 shell-4) was another 1 cm concentric contraction from the previous one. The most inner part located 4–4.5 cm from the outer contour of GTV covered the center of GTV (GTV_center_). Figure 1 illustrates these regions in three standard orthogonal planes and 3D display of OARs for patient 1.

**Table 1:** Patient, tumor, and treatment characteristics

No	Age (Diagnosis/RT)	Primary tumor site	Type of resection	Genetic mutation	TKIs therapy	Indication for RT	Tumor size before RT
1	62/67	Small intestine	R_0_	NA	Imatinib 24 M /sunitinib 36 M	Progression on TKIs resistance and unresectable	18.0 cm
2	50/55	Jejunum	R_0_	C-kit(E11)p.L576P, C-kit(E13)p.V654A	Imatinib 32 M /sunitinib 12 M/regorafenib 6 M	Progression on TKIs resistance and unresectable	17.2 cm
3	56/60	Ileum	R_0_	NA	Sunitinib 42 M	Progression on TKIs resistance and unresectable	20.0 cm
4	50/51	Duodenum	R_0_	NA	Imatinib 4 M/sunitinib 5 M	Progression on TKIs resistance and unresectable	12.0 cm
5	51/58	Jejunum	R_0_	C-kit(E11), C-kit(E17)p.Y823D	Imatinib 78 M/sunitinib 9 M	Progression on TKIs resistance and unresectable	17.0 cm
6	57/60	Small intestine	R_0_	NA	Imatinib 21 M/sunitinib 18 M	Progression on TKIs resistance and unresectable	14.5 cm
7	39/49	Small intestine	R_0_	C-kit(E17)p.N822K	Imatinib 55 M/sunitinib 26 M/regorafenib 2 M	Progression on TKIs resistance and unresectable	16.5 cm

No.: patient number; R0: microscopically margin-negative resection. M: the length of time (months) that patients take TKIs. Tumor size before RT: the maximum diameter of tumor in the diagnostic CT imaging

### Treatment planning

In this study, a Con-IMRT plan and four SIB-IMRT plans were generated in a dual-arc modality with 6 MV photon beams and calculated by high resolution collapsed cone convolution algorithm by Raystation™ treatment planning system version 4.7.5 (Raysearchlabs, Stockholm, Sweden) for each patient. All plans were created by an experienced medical physicist and reviewed by a senior medical physicist.

All settings were kept the same for different plans of the same patient except for the dose-gradient of target volumes. Table 2 shows the dose goals used for planning in this study. The prescription dose for PTV and shell-1 were both set to 50 Gy in 25 fractions. The dose of shell-2 was set to 55 Gy (110% of prescription dose), while the dose of shell-3, shell-4 and GTV_center_ were boosted to different dose in four SIB-IMRT groups (the subscript of the plan represents the escalation-dose gradient, see details in Table 2). For comparison, the Con-IMRT plan with 50 Gy for PTV and GTV were generated in the same beam arrangement.

**Table 2:** Escalation dose gradient for ellipsoidal regions inside the GTV and dose constraints for OARs

Region	Con-IMRT	SIB_10_-IMRT	SIB_15_-IMRT	SIB_20_-IMRT	SIB_25_-IMRT
Shell-1	50.0 Gy (100%) D1cc < 52.0 Gy	50.0 Gy (100%) D1cc < 52.0 Gy	50.0 Gy (100%) D1cc < 52.0 Gy	50.0 Gy (100%) D1cc < 52.0 Gy	50.0 Gy (100%) D1cc < 52.0 Gy
Shell-2	50.0 Gy (100%) D1cc < 52.0 Gy	55.0 Gy (110%) D1cc < 57.0 Gy	55.0 Gy (110%) D1cc < 57.0 Gy	55.0 Gy (110%) D1cc < 57.0 Gy	55.0 Gy (110%) D1cc < 57.0 Gy
Shell-3	50.0 Gy (100%) D1cc < 52.0 Gy	60.0 Gy (120%) D1cc < 62.0 Gy	62.5 Gy (125%) D1cc < 65.0 Gy	65.0 Gy (130%) D1cc < 67.0 Gy	67.5 Gy (135%) D1cc < 70.0 Gy
Shell-4	50.0 Gy (100%) D1cc < 52.0 Gy	65.0 Gy (130%) D1cc < 67.0 Gy	70.0 Gy (140%) D1cc < 72.0 Gy	75.0 Gy (150%) D1cc < 77.0 Gy	80.0 Gy (160%) D1cc < 82.0 Gy
GTV_center_	50.0 Gy (100%) D1cc < 52.0 Gy	70.0 Gy (140%)	77.5 Gy (155%)	85.0 Gy (170%)	92.5 Gy (185%)
intestine	D195cc < 43 Gy, D1cc < 50 Gy				
rectume	V45Gy < 45%, V40Gy < 60%				
bladder	V45Gy < 45%, V40Gy < 60%				
Femeral heads	V40Gy < 4%				
Ring1	Dmax < 48 Gy				
Ring2	Dmax < 40 Gy				

SIB_10_-IMRT is optimized by 10% dose-escalation gradient

SIB_15_-IMRT is optimized by 15% dose-escalation gradient

SIB_20_-IMRT is optimized by 20% dose-escalation gradient

SIB_25_-IMRT is optimized by 25% dose-escalation gradient

### Plan analysis and evaluation

For better comparison, all plans were normalized to 95% of the PTV covered by the prescription dose of 50 Gy and were analyzed using SPSS statistical analysis software package 23.0 (SPSS Inc., Armonk, NY). The Friedman test was performed among five groups with different escalation-dose gradients. Pairwise comparison between any two groups was performed when result of Friedman test was found to be significantly different. A *p* value < 0.05 was considered statistically significant.

#### The equivalent uniform dose indexes

To quantify the ability of multi-shell SIB-IMRT strategy to deliver high effective biological doses, the *EUD* based on DVH was used for GTV, OARs and NT structure [20–22]. The *EUD* was calculated by a MATLAB program from Hiram A. Gay’s paper [20].

$$EUD = \left( {\mathop \sum \limits_{i = 1} \left( {v_{i} \left( {D_{i} \frac{{\alpha /\beta + D_{i} /n_{f} }}{\alpha /\beta + 2}} \right)^{a} } \right)} \right)^{\frac{1}{a}}$$

where the parameter *a* was a negative value (− 10) for target volume and a positive value (1) for NT structure in this study, Di and vi data pairs were obtained from differential dose volume histogram of a given radiotherapy plan, Vi was the part of the target volume irradiated by a dose Di. ${n}_{f}$ was the number of fractions. The relative *EUD* (*rEUD*) was defined as the ratio of the *EUD* of GTV (*EUD*_*GTV*_) to that of NT structure (*EUD*_*NT*_). A higher *rEUD* value represented a higher therapeutic gain ratio and indicated a better dose escalation and higher effective biological dose for target volume, but a low dose for normal tissue.

#### Dose-volume histograms and irradiation for healthy tissues

Besides, *D*_*mean*_ (the mean dose), *D*_*1cc*_ (the received dose to 1cm^3^), *D*_*2cc*_, V_20_ (the percentage volume of the OAR receiving ≥ 20 Gy), V_30_, V_40_, V_50_ of OARs were compared between the Con-IMRT group and any SIB-IMRT group.

#### Conformity index and monitor unit

The conformity index (CI) of PTV and monitor unit (MU) were obtained and compared. A CI value closer to 1 indicated a more conformal dose distribution to PTV and a better normal tissue sparing [23].

$$\mathrm{CI}=\frac{{TV}_{RI}}{TV}\times \frac{{TV}_{RI}}{{V}_{RI}}$$

where the parameter ${TV}_{RI}$ was the target volume covered by the prescribed dose, $TV$ was the target volume, ${V}_{RI}$ was the volume covered by the prescribed dose.

## Results

### Evaluation of target volume in different plans

**Fig. 2:**
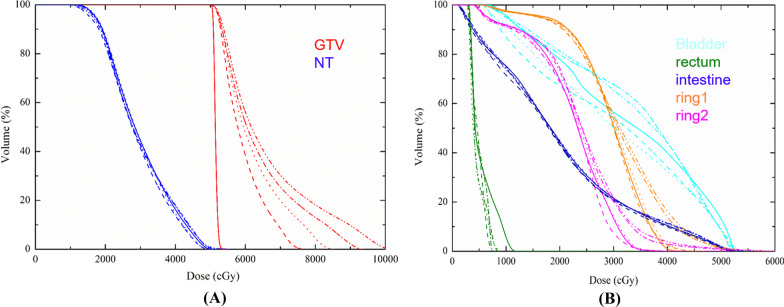
DVH comparisons between Con-IMRT (solid line) and SIB-IMRTs (the dash line is SIB_10_-IMRT, the dot line is SIB_15_-IMRT, the dash dot line is SIB_20_-IMRT plan, the dash dot dot line is SIB_25_-IMRT plan) **A** for GTV and NT structure; **B** for OARs, ring 1 and ring 2 structure

**Fig. 3:**
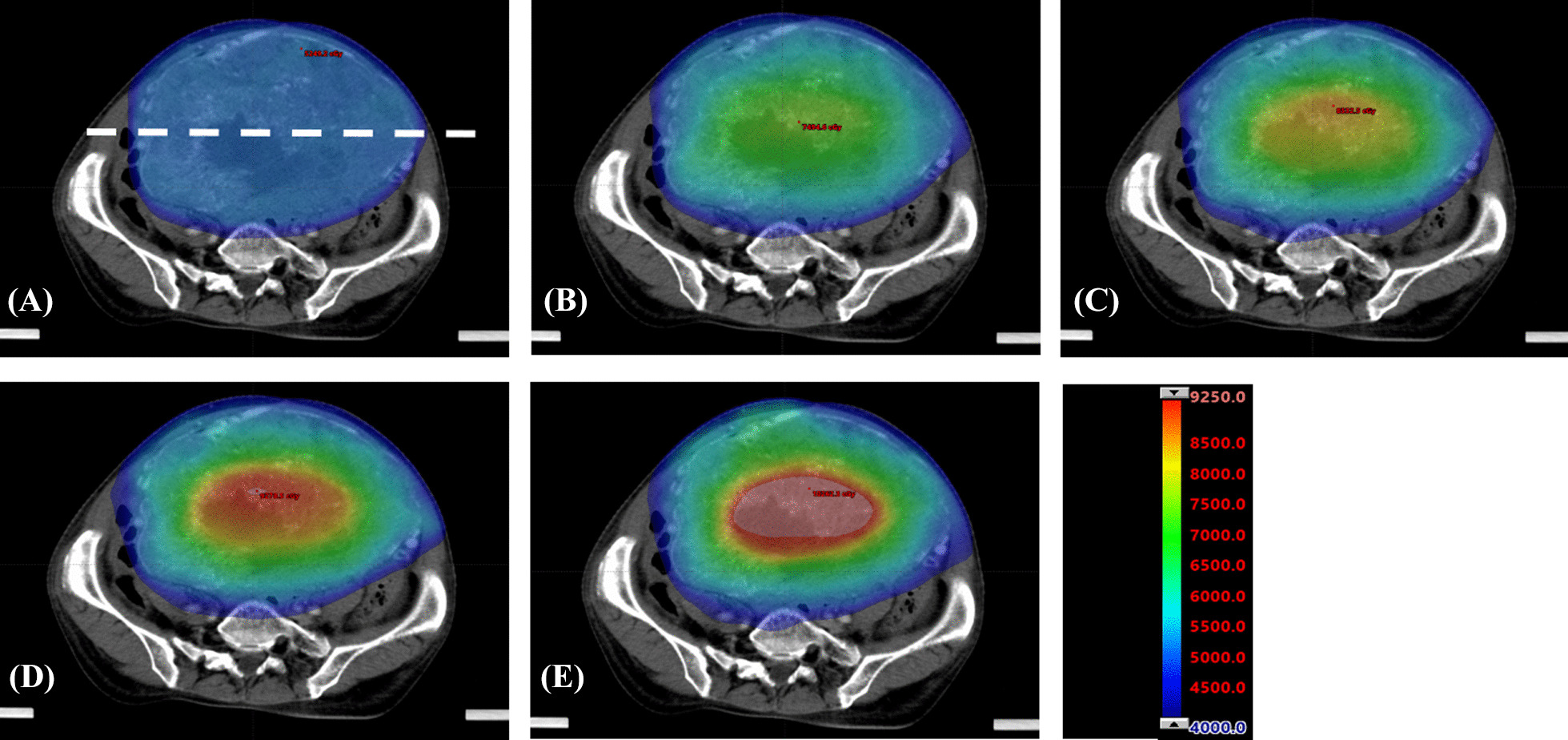
Difference in dose distributions in patient 1 between Con-IMRT plan and SIB-IMRT plans visualized in axial planes. **A** The axial plane in Con-IMRT plan, **B** the axial plane in SIB_10_-IMRT plan, **C** the axial plane in SIB_15_-IMRT plan, **D** the axial plane in SIB_20_-IMRT plan, and **E** the axial plane in SIB_25_-IMRT plan

**Fig. 4:**
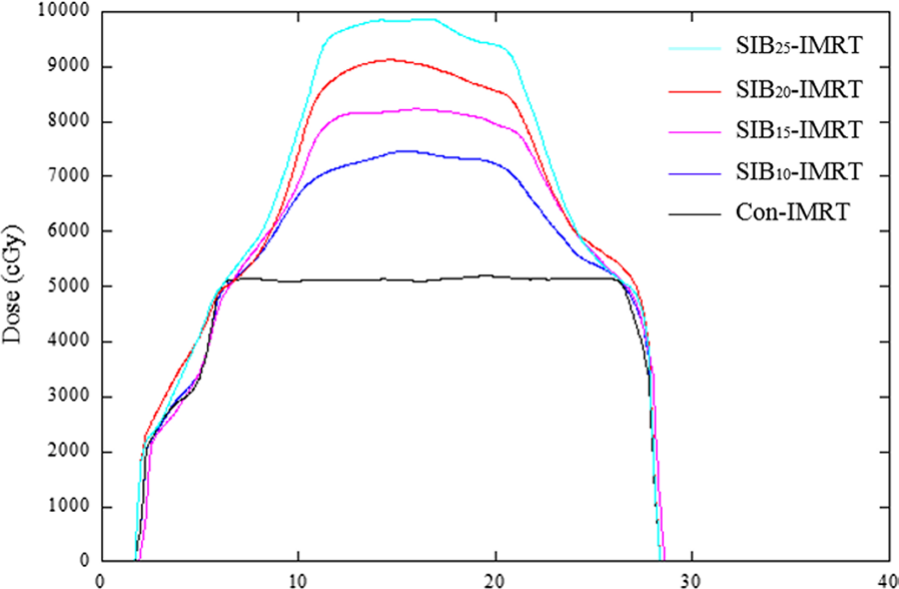
Comparison of dose profiles of different plans in patient 1. The black line is the Con-IMRT plan, the blue line is SIB_10_-IMRT plan, the magenta line is SIB_15_-IMRT plan, the red line is SIB_20_-IMRT plan, and the cyan line is SIB_25_-IMRT plan

Figure 2A showed an example of the DVH comparison of GTV and NT between the Con-IMRT plan and SIB-IMRT plans. Significant dose escalations were observed in GTV of the SIB-IMRT plans, while no significant dose difference in DVH information was found among NT of these plans. As shown in Fig. 3, The SIB-IMRT plans showed higher and more concentrated dose distribution of the target volume compared with the Con-IMRT plan. As shown in Fig. 3A, the dose profile was extracted along the dashed line of dose distribution in each plan. The comparison of profiles (Fig. 4) demonstrated that SIB-IMRT plans with higher *EUD*_*GTV*_ had a steeper dose gradient within GTV, while the dose profiles excluding PTV were nearly consistent with that of Con-IMRT plan. Obviously, the SIB-IMRT groups had higher *EUD*_*GTV*_ and *rEUD* than that of the Con-IMRT group (shown in Table 3). The Friedman test showed a significant difference in *EUD*_*GTV*_ and *rEUD* among the five groups (*P* < 0.05). Subsequently, the result of Pairwise comparisons showed that the *EUD*_*GTV*_ and *rEUD* of SIB-IMRT group were not significantly different from that of Con-IMRT group until the escalation-dose gradient was up to 20% of the prescription dose (For *EUD*_*GTV*_: Con-IMRT VS SIB_20_-IMRT, *P* = 0.004, Con-IMRT VS SIB_25_-IMRT, *P* = 0; For *rEUD*: Con-IMRT VS SIB_20_-IMRT, *P* = 0.004, Con-IMRT VS SIB_25_-IMRT, *P* = 0). However, there was no significant difference between any two SIB-IMRT groups optimized with different escalation-dose gradient, except that between the SIB_10_-IMRT group and SIB_25_-IMRT group (For *EUD*_*GTV*_, *P* = 0.041; For *rEUD*, *P* = 0.04). Moreover, with the increase of the dose-escalation gradient, the *rEUD* of different groups was found to become stable (SIB_20_-IMRT group and SIB_25_-IMRT group have the similar mean *rEUD*).

**Table 3:** Comparison of *EUDs* and *rEUDs* of different groups (mean and range)

Groups	*EUD*_*GTV*_(Gy)	*EUD*_*NT*_(Gy)	*rEUD*(Gy)
Con-IMRT	51.62 (50.98–52.42)	16.47 (12.31–25.55)	3.31 (2.02–4.26)
SIB_10_-IMRT	58.29 (56.41–60.03)	**16.21** (12.25–24.49)	3.77 (2.44–4.90)
SIB_15_-IMRT	59.97 (57.38–62.49)	16.41 (12.01–25.41)	3.85 (2.46–5.02)
SIB_20_-IMRT	61.30 (58.34–64.41)	16.57 (12.49–25.48)	3.88 (2.53–5.05)
SIB_25_-IMRT	**62.89** (59.12–66.56)	16.85 (12.73–25.45)	**3.90** (2.62–5.13)
*p*-values	< 0.001*	0.006**	< 0.001***

Bold represented the optimal values of parameters. It displayed the trend of each parameter more plainly

*The significant differences of Pairwise comparisons for *EUD*_*GTV*_: Con-IMRT VS SIB_20_-IMRT, *P* = 0.004; Con-IMRT VS SIB_25_-IMRT, *P* = 0; SIB_10_-IMRT VS SIB_25_-IMRT, *P* = 0.041

**The significant differences of Pairwise comparisons for *EUD*_*NT*_: SIB_10_-IMRT VS SIB_25_-IMRT, *P* = 0.007

***The significant differences of Pairwise comparisons for *rEUD*: Con-IMRT VS SIB_20_-IMRT, *P* = 0.004; Con-IMRT VS SIB_25_-IMRT, *P* = 0; SIB_10_-IMRT VS SIB_25_-IMRT, *P* = 0.040

### Evaluation of healthy tissues in different plans

Friedman test showed significant differences in *EUD*_*NT*_, the *EUD* of bladder (*EUD*_*bla*_), the *EUD* of intestine (*EUD*_*ins*_) and *D*_*mean*_ of bladder among the five groups (*P* < 0.05)_._ Subsequently, significant differences between SIB_10_-IMRT group and SIB_25_-IMRT group (*P* < 0.05) were found in the Pairwise Comparisons. Tables 4, 5, 6 and 7 summarized the comparisons of dosimetry parameters and EUDs for OARs. With the increase of escalation-dose gradient, the DVH metrics (V_20_, V_30_, V_40_, V_50_, *D*_*mean*_, *D*_*1cc*_, *D*_*2cc*_) and *EUDs* slightly decreased first and then increased. Most DVH indexes of SIB_10_-IMRT group were even lower than those of the Con-IMRT group. An example of the DVH comparison for healthy tissues was shown in Fig. 2B. For the bladder, there were lower irradiated volumes for SIB-IMRT plans compared with that for the Con-IMRT plan, except the volumes irradiated with a dose level from 2000 to 4300 cGy in SIB_20_-IMRT plan and SIB_25_-IMRT plan. For the rectum, all SIB-IMRT plans provided lower irradiated volumes compared with the Con-IMRT plan. For intestine, five plans provided similar irradiated volumes as shown in DVH information. For ring 1 structure, the SIB_25_-IMRT plan provided the highest irradiated volume, followed by SIB_20_-IMRT, then followed by Con-IMRT and SIB_15_-IMRT, while the SIB_10_-IMRT plan provided the lowest irradiated volume. The DVHs for ring 2 structure was similar to that for ring 1 structure.

**Table 4:** Summary of DVH-based analysis for the bladder in five groups

Groups	V_20_(%)	V_30_(%)	V_40_(%)	V_50_(%)	*D*_*1cc*_ (cGy)	*D*_*2cc*_ (cGy)	*D*_*mean*_(cGy)	*EUD*_*bla*_(cGy)
Con-IMRT	96.10 (77.90–100.0)	76.10 (42.00–97.10)	43.42 (15.70–66.32)	11.50 (0.90–31.90)	5154.12 (5010.90–5246.00)	**5088.70** (4966.30–5207.70)	3747.82 (3064.30–4360.10)	36.14 (28.11–42.96)
SIB_10_-IMRT	93.95 (67.20–100.0)	75.25 (41.70–95.70)	**40.83** (15.20–64.76)	**8.79** (0.70–26.20)	**5147.27** (5003.20–5418.50)	5102.40 (4923.50–5335.80)	3663.87 (3020.60–4304.50)	35.28 (27.57–42.32)
SIB_15_-IMRT	**91.23** (70.40–100.0)	**74.02** (35.80–95.30)	41.39 (12.90–64.91)	9.43 (1.20–28.30)	5208.35 (5044.00–5505.10)	5147.88 (4971.00–5416.00)	**3652.37** (2817.30–4340.40)	**35.20** (25.95–42.81)
SIB_20_-IMRT	96.33 (79.90–100.0)	77.75 (51.10–97.00)	43.28 (13.20–65.28)	9.36 (0.70–31.40)	5197.47 (5000.40–5435.70)	5128.37 (4927.60–5370.40)	3755.48 (3146.60–4378.60)	36.03 (28.39–43.26)
SIB_25_-IMRT	96.82 (81.30–100.0)	82.78 (65.50–99.40)	44.89 (16.40–67.25)	9.89 (1.00–30.60)	5225.93 (5027.20–5432.70)	5153.85 (4961.40–5375.60)	3833.98 (3348.70–4386.40)	36.76 (30.44–43.28)
*p*-values	0.293	0.139	0.053	0.033*	0.220	0.066	0.034**	0.043***

Bold represented the optimal values of parameters. It displayed the trend of each parameter more plainly

*EUD*_*bla*_: the *EUD* of bladder

*The lowest p-value of Pairwise comparisons for V_50_ (%): Con-IMRT VS SIB_10_-IMRT, *P* = 0.081

**The significant differences of Pairwise comparisons for *D*_*mean*_: SIB_10_-IMRT VS SIB_25_-IMRT, *P* = 0.019

***The significant differences of Pairwise comparisons for *EUD*_*bla*_: SIB_10_-IMRT VS SIB_25_-IMRT, *P* = 0.035

**Table 5:** Summary of DVH-based analysis for the rectum in in five groups

Groups	V_20_(%)	V_30_(%)	V_40_(%)	V_50_(%)	*D*_*1cc*_ (cGy)	*D*_*2cc*_ (cGy)	*D*_*mean*_(cGy)	*EUD*_*rec*_(cGy)
Con-IMRT	62.60 (0.0–100.0)	42.86 (0.0–97.20)	**24.78** (0–77.80)	12.05 (0.0–49.50)	3511.25 (1016.50–5275.20)	3416.82 (943.10–5256.60)	2747.20 (541.90–4575.20)	27.88 (5.44–49.04)
SIB_10_-IMRT	64.24 (0.0–100.0)	41.61 (0.0–96.30)	24.95 (0.0–80.60)	11.65 (0.0–49.70)	3534.83 (670.50–5497.60)	3433.68 (638.90–5464.30)	2723.33 (469.00–4684.50)	27.82 (3.61–50.48)
SIB_15_-IMRT	**58.78** (0.0–100.0)	40.71 (0.0–96.40)	25.98 (0.0–86.10)	**11.43** (0.0–49.80)	**3473.12** (746.20–5487.80)	**3399.35** (691.10–54,600)	**2669.15** (447.00–4658.00)	**27.50** (3.93–50.48)
SIB_20_-IMRT	68.66 (0.0–100.0)	**40.63** (0.0–99.00)	25.51 (0.0–82.50)	12.17 (0.0–52.00)	3528.57 (712.60–5616.80)	3445.38 (4696.00–5564.00)	2761.67 (487.00–4722.00)	28.04 (3.88–51.29)
SIB_25_-IMRT	74.50 (0.0–100.0)	43.53 (0.0–96.10)	25.37 (0.0–82.00)	12.22 (0.0–53.00)	3572.35 (758.80–5562.40)	3489.28 (715.80–5506.00)	2812.43 (467.50–4692.40)	28.53 (3.97–50.88)
*p*-values	0.537	0.844	0.938	0.525	0.525	0.592	0.070	0.078

Bold represented the optimal values of parameters. It displayed the trend of each parameter more plainly

*EUD*_*rec*_: the *EUD* of rectum

**Table 6:** Summary of DVH-based analysis for the intestine in five groups

Groups	V_20_(%)	V_30_(%)	V_40_(%)	V_50_(%)	*D*_*195cc*_ (cGy)	*D*_*1cc*_ (cGy)	*D*_*2cc*_ (cGy)	*D*_*mean*_(cGy)	*EUD*_*ins*_(cGy)
Con-IMRT	28.19 (8.70–46.80)	12.77 (2.80–21.60)	5.97 (0.50–10.4)	1.12 (0.0–2.60)	**2440.40** (32.61–4430.70)	5133.89 (4918.40–5316.50)	4876.87 (3731.20–5291.10)	1425.07 (533.10–2017.30)	30.25 (21.83–33.77)
SIB_10_-IMRT	**27.24** (8.70–43.10)	**12.41** (2.70–20.50)	**5.72** (0.50–9.90)	**0.97** (0.0–2.10)	2485.29 (32.04–4361.40)	**5113.31** (4710.40–5348.60)	**4841.24** (3664.40–5291.50)	**1383.79** (530.50–1953.60)	**29.59** (21.09–33.44)
SIB_15_-IMRT	27.37 (9.30–45.0)	12.67 (2.80–21.60)	5.83 (0.60–10.10)	1.07 (0.0–2.30)	2441.37 (32.21–4387.50)	5170.90 (4897.70–5403.80)	4905.77 (3701.90–5338.90)	1404.76 (537.20–2004.70)	30.12 (22.32–33.58)
SIB_20_-IMRT	28.01 (8.90–43.90)	12.84 (2.80–20.90)	5.94 (0.50–11.30)	1.10 (0.0–2.20)	2439.43 (32.228–451.80	5161.34 (4723.80–5569.50)	4884.20 (3689.60–5508.40)	1422.64 (534.80–2014.10)	30.09 (21.16–34.47)
SIB_25_-IMRT	27.19 (9.50–43.6)	12.59 (3.10–21.00)	6.04 (0.60–11.60)	1.21 (0.0–2.70)	2497.93 (32.10–4541.00)	5233.63 (4869.70–5617.20)	4945.10 (3670.00–5523.90)	1426.23 (543.40–2018.70)	30.37 (22.14–34.48)
*p*-values	0.667	0.798	0.683	0.047*	0.869	0.171	0.296	0.115	0.007**

Bold represented the optimal values of parameters. It displayed the trend of each parameter more plainly

*EUD*_*ins*_: the *EUD* of intestine

*The lowest p-value of Pairwise comparisons for V_50_ (%): SIB_10_-IMRT VS SIB_25_-IMRT, *P* = 0.068

**The significant differences of Pairwise comparisons for *EUD*_*ins*_: Con-IMRT VS SIB_10_-IMRT, *P* = 0.007; SIB_10_-IMRT VS SIB_25_-IMRT, *P* = 0.023

**Table 7:** Summary of DVH-based analysis for the femoral heads in five groups

Groups	V_20_(%)	V_30_(%)	V_40_(%)	V_50_(%)	*D*_*1cc*_ (cGy)	*D*_*2cc*_ (cGy)	*D*_*mean*_(cGy)	*EUD*_*fh*_(cGy)
Con-IMRT	33.06 (7.70–63.30)	4.82 (0.50–12.70)	0.56 (0.0–1.50)	–	3558.22 (2963.30–4447.30)	3390.52 (2888.80–4203.90)	1660.00 (1310.80–2154.50)	16.76 (12.02–20.62)
SIB_10_-IMRT	**31.54** (7.40–49.90)	4.06 (0.00–10.90)	**0.20** (0.0–0.50)	–	**3328.12** (2753.50–4028.70)	**3187.46** (2621.30–3835.30)	**1670.06** (1297.00–2039.70)	**16.05** (11.53–19.98)
SIB_15_-IMRT	32.90 (8.80–49.80)	**3.98** (0.20–10.90)	0.26 (0.0–0.90)	–	3347.78 (2845.50–41,280)	3208.44 (2710.60–3983.00)	1671.48 (1309.80–2026.80)	16.14 (11.99–20.31)
SIB_20_-IMRT	32.82 (8.00–58.80)	4.84 (0.20–12.90)	0.22 (0.0–0.70)	–	3385.56 (2912.80–4118.30)	3246.40 (2758.80–3945.70)	1688.14 (1311.40–2111.90)	16.36 (11.98–20.25)
SIB_25_-IMRT	36.26 (8.00–58.80)	5.54 (0.10–9.90)	0.48 (0.0–1.40)	–	3509.12 (2816.40–4183.60)	3374.44 (2699.50–3374.40)	1764.54 (1314.50–2211.90)	16.91 (12.06–20.77)
*p*-values	0.654	0.246	0.171	–	0.075	0.042*	0.231	0.126

Bold represented the optimal values of parameters. It displayed the trend of each parameter more plainly

*EUD*_*fh*_: the *EUD* of femoral heads

*The lowest *p*-values of Pairwise comparisons for *D*_*2cc*_: Con-IMRT VS SIB_10_-IMRT, *P* = 0.093; Con-IMRT VS SIB_15_-IMRT, *P* = 0.093

### CIs and MUs of different plans

Table 8 listed CIs and MUs of different plans. There was a slight change of the mean CIs among the rest groups except for that between the SIB_10_-IMRT group and SIB_25_-IMRT group (*P* < 0.05). Although the mean MUs (534–721) increased along with the dose escalation in SIB-IMRT groups, no statistically significant differences were found until the dose gradient was increased to 25%.

**Table 8:** Comparison of CIs and MUs in five groups

Groups	CIs	MUs
Con-IMRT	0.92 (0.909–0.938)	534.10 (365.0–772.0)
SIB_10_-IMRT	**0.93** (0.904–0.936)	565.30 (375.0–750.0)
SIB_15_-IMRT	0.92 (0.899–0.935)	626.60 (426.0–784.0)
SIB_20_-IMRT	0.92 (0.896–0.932)	670.30 (452.0–838.0)
SIB_25_-IMRT	0.92 (0.902–0.926)	721.10 (495.0–832.0)
*p*-values	0.006*	< 0.001**

Bold represented the optimal values of parameters. It displayed the trend of each parameter more plainly

*The significant difference of Pairwise comparisons for *CIs*: SIB_10_-IMRT VS SIB_25_-IMRT, *P* = 0.01

**The significant differences of Pairwise comparisons For *MUs*: Con-IMRT VS SIB_25_-IMRT, *P* = 0.013; SIB_10_-IMRT VS SIB_20_-IMRT, *P* = 0.023; SIB_10_-IMRT VS SIB_25_-IMRT, *P* = 0.001

## Discussion

In recent years, radiotherapy has been beneficial to the management of GISTs [4, 8, 9]. The result has shown that even short-term radiation therapy can effectively alleviate local symptoms, and its toxic and side effects for the localized progression or metastasis of GISTs is acceptable [24, 25]. Radiotherapy could play a role in the treatment of GISTs, either as an adjuvant therapy or a definitive treatment with or without a TKI [7, 8, 26]. But in previous studies [7, 9, 27], the tumor was usually under control for only a few months because the total bioequivalent doses were 30–50 Gy by conventional fractionation. In prospective study of Joensu et al. [27], metastases were treated with external beam radiotherapy using either conformal 3D planning or intensity modulated radiotherapy and conventional fractionation to a cumulative PTV dose of 30–40 Gy. Only 2 of 25 GISTs patients achieved partial remission after conventional radiotherapy. There are two main reasons. Firstly, GIST is a most common sarcoma in the gastrointestinal tract. It is relatively radio-resistant to conventional dose regimens perhaps due to the histological relation to soft-tissue sarcoma which has a relatively slow clinical responsiveness to radiation therapy [28]. Secondly, tumor size is one of the most important factors to predict the malignant potential of GISTs. The larger the tumor, the larger the hypoxia area. Especially in the center of the target volume, an inadequate radiation dose will result in poor prognosis. Therefore, a higher equivalent uniform dose and a heterogeneous distribution seems to be needed in GTV for LADR-GIST.

The SBRT or SIB-IMRT is the commonly used technique for delivering high and heterogeneous dose to hypoxic tumor. However, SBRT technique is not suitable for a huge tumor, especially for the tumor located in abdomen because of the radiosensitivity of OARs. In modern radiation therapy techniques, unconventional approaches have also been allowed to enable a large tumor to receive highly heterogeneous doses. Spatial modulation of megavoltage therapy beams, commonly referred to as spatially fractionated radiation therapy (SFRT) (e.g., “Lattice” radiation therapy), is a novel technique which purposefully enables the treated tumor to receive high degree of heterogeneous doses. Developed from the previous high-dose GRID radiotherapy, “Lattice” allows for localized 3D high-dose array within the tumor. This method provides lattice vertices with highly concentrated hot-spot doses, while provides rapid decreased doses between lattices, resulting in a periodic three-dimensional peak-to-valley dose distribution. It has shown a promising future in clinical studies as a method to improve treatment response of advanced and bulky tumors. However, this technique has been applied by only a few centers in clinical practice, more wide and effective application are still needed [29–31]. Fortunately, it has been proven that an appropriate SIB-IMRT strategy is able to deliver high biologically effective dose to large tumors without significant increase of healthy tissue irradiation [32–35]. In the study of Nomiya et al. [35], the dose for central of target area was boosted to 120% of the prescription dose by SIB-IMRT plan without upper dose constraint. Significant tumor regression was observed after radiotherapy, and no related toxic reactions were found. Savino et al. [36] proposed a modified SIB-IMRT for a patient having a huge chordoma with large swelling infiltrating in the right gluteal region and the ipsilateral thigh root. The proposed SIB-IMRT delivered 200% of PTV prescription dose to boost target volume (BTV) with an acceptable increased irradiation dose for healthy tissues surrounding PTV. In our previous study [10], three patients with LADR-GIST were treated by SIB-IMRT plans, and the radiation dose for GTV-center was escalated up to 125% of the prescribed dose in PTV. During follow-up, three patients were assessed as partial response based on Choi criteria [11]. These results have indicated that a heterogeneous or stereotactic-like distribution based on an appropriate SIB-IMRT modality has advantages in management of LADR-GIST.

Undoubtedly, it is a challenge to design an appropriate SIB-IMRT modality for LADR-GIST. In general, LADR-GIST has two key characteristics. One is that the closer to the center of the target area, the more severe the hypoxia is; the other is that the tumor is surrounded by many OARs. Based on these characteristics, the escalation dose multi-shell SIB-IMRT planning strategy was proposed in this study. It was different from the conventional SIB-IMRT technique in which the dose-escalation was simultaneously delivered to different target volumes [37, 38]. In this study, only one target volume GTV was intentionally divided into several parts from outside to inside by multi-shell structures. In previous studies, the shell-structures around target volume have been commonly used in SBRT plans to adjust the dose gradient. The number and width of shells should be adjusted according to the target volume, clinical justifications, and planning goals. The use of a shell structure of 1–3 mm was recommended for limiting the high dose conformity. Besides, the two-shell structure was recommended with a distance between each other of 5–10 mm for the optimization of the prescription dose conformity, and a distance between each other of 15–20 mm for the optimization of the proximate dose fall-off. Additionally, a shell structure of 30–50 mm was suggested for limiting the skin doses and hot spots outside the proximate target region [18, 39]. Those studies reported that a steeper dose fall-off could be achieved with the increase of the number of shells. However, the quality of the plan exhibits relatively small improvement when the number of the shells was more than 6 [18, 19]. Different from the previous studies, four shells inside target volume were utilized for dose escalation in this study. The inner shells (shell-3, shell-4, GTV_center_) were utilized for delivering as much radiation dose as possible to the central of GTV to improve the response of tumor. The maximum dose in GTV_center_ could be escalated up to 197% of the prescription dose (Fig. 4). It was worth noting that the maximum EUD_GTV_ could be accumulated to 62.9 Gy (ranging from 59.1 to 66.6 Gy) (Table 3) in the SIB-IMRT groups, which was approximately 122% of that in the Con-IMRT group. This stereotactic-like dose based on SIB-IMRT strategy formed a “hot core” in the central of target volume (Fig. 3), and induced a higher rate of tumor cell apoptosis in bulky and hypoxic tumors. The exterior shells (shell-1, shell-2) played a major role in avoiding higher radiation dose for OARs, the radiation dose of shell-1 and shell-2 were set to 100%, 110% of the prescribed dose in GTV, respectively. As shown in Tables 3, 4, 5, 6 and 7, the delivered target volume was increased to a significant higher dose without any compromise of healthy tissue sparing. The size of shell-1 was determined by actual position of the target volume and OARs. In this study, shell-1 was defined as 1–1.5 cm concentric contraction from the outer contour of GTV in view of the overlap region between OARs and GTV. Compared with the shell-structure around PTV, the number and width of shells inside PTV was more dependent on the target size. For the same target volume, the number of shells was inversely proportional to the width of shells, that is, the smaller the width, the more the number of shells. A steeper dose escalation (higher EUD_GTV_) could be achieved with more shells or higher escalation-dose gradient. But at the same time, the risk of dose spillover will increase (in Fig. 4). Therefore, the setting of shells and escalation-dose gradient would lead to a significant trade-off between the EUD_GTV_ and the healthy tissue dose. In this study, the setting of shells and the escalation-dose gradient were the optimal solution when the dose gradient reached 20% of the prescription dose. If wider shells were provided for plans with higher dose gradient, the contribution of higher dose gradient to EUD_GTV_ will be weakened by the reducing of the number of shells. Therefore, an optimal therapeutic gain ratio can be achieved by a reasonable shell-structure and escalation dose scheme.

In order to quantitatively evaluate the therapeutic gain ratio of radiotherapy, the *rEUD* was proposed. As shown in Table 3, all SIB-IMRT groups have better *EUD*_*GTV*_ and *rEUD* compared with the Con-IMRT group, the *EUD*_*GTV*_ and *rEUD* were improved when escalation-dose gradient was increased, and the improvement became significant when the escalation-dose gradient reached 20% of the prescription dose (*P* < 0.05). Along with the increase of escalation-dose gradient, the value of *rEUD* tended to be stable (the *rEUD* of SIB_20_-IMRT group was similar to that of SIB_25_-IMRT group). The reasons may be as follows, firstly, shell-1 and shell-2 which accounted for nearly 60% of the GTV volume (shown in Fig. 1) received almost the same radiation dose in four SIB-IMRT groups (shown in Table 2); secondly, the difference of escalation-dose gradient was just 5% of prescription dose between any two neighboring SIB-IMRT groups; thirdly, the *EUD*_*NT*_ increased along with the increase of the escalation-dose gradient. As shown in Tables 4, 5, 6 and 7, the DVH comparison results in OARs demonstrated that a reasonable utilization of shell structure enabled SIB-IMRT plan to generate a required escalation-dose gradient. In our study, the DVH metrics and EUDs for OARs showed a trend of slightly decreasing first and then increasing with the increase of escalation-dose gradient (see Fig. 2B and Tables 4, 5, 6 and 7). There were no significant differences of DVH parameters for OARs between the Con-IMRT group and any SIB-IMRT group. However, significant differences were found in *EUD*_*NT*_, *EUD*_*bla*_, *EUD*_*ins*_*,* CI and *D*_*mean*_ of bladder between the SIB_10_-IMRT group and the SIB_25_-IMRT group (*P* < 0.05). In addition, most DVH indexes of the SIB_10_-IMRT group were found to be even lower than those of the Con-IMRT group (see Tables 4, 5, 6 and 7). The results implied that the SIB_10_-IMRT group had the advantage of OAR’s sparing caused by the relaxation of GTV upper dose constraint in SIB-IMRT plans in which the relative weight of all other constraints increased [10]. However, the advantage was weakened even offset with the increase of escalation-dose gradient. Thus, when the escalation-dose gradient reached 25% of the prescribed dose, the difference became significant compared with the SIB_10_-IMRT group. It was implied that 20% of the prescribed dose may be the optimal escalation-dose gradient, which can provide a high therapeutic gain ratio without overdosing the OARs compared with the Con-IMRT group. It was also safe and effective to set the escalation-dose gradient to 25% of the prescribed dose in this target volume, while the DVH metrics of OARs tended to increase. These results also demonstrated that the escalation-dose gradient delivered to target volume could not be increased limitlessly. Given the above, the proposed multi-shell SIB-IMRT strategy is able to provide high escalation dose to a LADR-GIST while maintaining the similar dose level for OARs. The detailed escalation dose scheme must be synthetically considered together with tumor size, overlap region between the tumor and OARs, prescribed dose, and shell-structure and so on.

Nonetheless, there are still several limitations in this study. Firstly, the sample size was small, and only seven patients were studied because of the rigid inclusion criteria. Maybe 20% of the prescribed dose is not the optimal dose gradient as more samples are included in the future. However, it would not affect the direction provided in this study, that is, an optimal therapeutic gain ratio can be achieved by reasonable shell-structures and escalation dose scheme. In our study, the results indicated that initial optimization dose gradient could be set to 20% of the prescribed dose. The final optimization dose gradient should be adjusted according to the actual situation. A large sample size is required and further investigation need to be implemented for the optimal dose gradient. Secondly, the variation of shell-structure was not intensively investigated, because our focus was on the exploration of dose gradient regimen. The selection of shell parameters would be an attractive topic, because it is dependent on the target volume and OARs. Thirdly, it is only a dosimetry comparison, which means further clinical trials is still required.

## Conclusion

The proposed multi-shell SIB-IMRT strategy is safe and reliable in dosimetry. It is able to provide a feasible scheme for patients having LADR-GIST by providing high dose distribution for the center of a large tumor. In our study, the dosimetry evaluation demonstrated that it is safe and optimal to apply 20% of the prescribed dose as the escalation-dose gradient for LADR-GISTs. However, the scheme of delivering 20% of the prescribed dose gradient should be further evaluated in future clinical study.

## Data Availability

The datasets used and analyzed during the current study are available from the corresponding author on reasonable request.

## Abbreviations

LADR-GIST: Large locally advanced drug-resistant gastrointestinal stromal tumor
SIB-IMRT: Simultaneous integrated boost intensity-modulated modality
GTV: Gross tumor volume
GTV_center_: Center of gross tumor volume
PTV: Planning target volume
CTV: Clinical target volume
NT: Normal tissue
Con-IMRT: Conventional IMRT technique
*EUD*: Equivalent uniform dose
*rEUD*: Relative equivalent uniform dose
DVH: Dose volume histogram
OARs: Organs at risk
CI: Conformity index
MU: Monitor unit
*EUD*_*GTV*_: Equivalent uniform dose of gross tumor volume
*EUD*_*NT*_: Equivalent uniform dose of normal tissue
GISTs: Gastrointestinal stromal tumors
KIT: Tyrosine kinase receptor
PDGFRA: Platelet-derived growth factor receptor alpha
TKIs: Tyrosine kinase inhibitors
SBRT: Stereotactic body radiotherapy
*EUD*_*bla*_: Equivalent uniform dose of bladder
*E**UD*_*rec*_: Equivalent uniform dose of rectum
*EUD*_*ins*_: Equivalent uniform dose of intestine
*E**UD*_*fh*_: Equivalent uniform dose of femoral heads
SFRT: Spatially fractionated radiation therapy
BTV: Boost target volume

## References

[1] Joensuu H, Hohenberger P, Corless CL (2013). Gastrointestinal stromal tumour. Lancet.

[2] Akahoshi K, Oya M, Koga T, Shiratsuchi Y (2018). Current clinical management of gastrointestinal stromal tumor. World J Gastroenterol.

[3] Nishida T, Blay JY, Hirota S, Kitagawa Y, Kang YK (2016). The standard diagnosis, treatment, and follow-up of gastrointestinal stromal tumors based on guidelines. Gastric Cancer.

[4] Ozkan EE (2018). Radiotherapy for gastrointestinal stromal tumors. Chin Med J.

[5] Network. NCC. NCCN clinical practice guidelines in oncology: soft tissue sarcoma, version 2.2020. 2020.

[6] Casali PG, Blay JY, Abecassis N, Bajpai J, Bauer S, Biagini R (2021). Gastrointestinal stromal tumours: ESMO-EURACAN-GENTURIS clinical practice guidelines for diagnosis, treatment and follow-up. Ann Oncol.

[7] Yang P-C, Guo J-C (2018). Radiotherapy as salvage treatment after failure of tyrosine kinase inhibitors for a patient with advanced gastrointestinal stromal tumor. J Cancer Res Prac.

[8] Knowlton CA, Brady LW, Heintzelman RC (2011). Radiotherapy in the treatment of gastrointestinal stromal tumor. Rare Tumors.

[9] Cuaron JJ, Goodman KA, Lee N, Wu AJ (2013). External beam radiation therapy for locally advanced and metastatic gastrointestinal stromal tumors. Radiat Oncol.

[10] Li L, Yi X, Cui H, Zhao X, Dang J, Jiang Q (2020). Simultaneous integrated boost intensity-modulated radiotherapy for locally advanced drug-resistant gastrointestinal stromal tumors: a feasibility study. Front Oncol.

[11] Choi H, Charnsangavej C, Faria SC, Macapinlac HA, Burgess MA, Patel SR (2007). Correlation of computed tomography and positron emission tomography in patients with metastatic gastrointestinal stromal tumor treated at a single institution with imatinib mesylate: proposal of new computed tomography response criteria. J Clin Oncol.

[12] Challapalli A, Carroll L, Aboagye EO (2017). Molecular mechanisms of hypoxia in cancer. Clin Transl Imag.

[13] Begg K, Tavassoli M (2020). Inside the hypoxic tumour: reprogramming of the DDR and radioresistance. Cell Death Discov.

[14] Zaghloul M. Simultaneous integrated boost-intensity modulated radiotherapy (SIB-IMRT) for the whole pelvis did not lead to significantly higher toxicity rates than prostate-only IMRT in prostate cancer patients. Clin Oncol. 2016.

[15] Feng CH, Hasan Y, Kopec M, Al-Hallaq HA (2016). Simultaneously integrated boost (SIB) spares OAR and reduces treatment time in locally advanced cervical cancer. J Appl Clin Med Phys.

[16] Voet PW, Dirkx ML, Breedveld S, Heijmen BJ (2013). Automated generation of IMRT treatment plans for prostate cancer patients with metal hip prostheses: comparison of different planning strategies. Med Phys.

[17] Sharfo AW, Voet PW, Breedveld S, Mens JW, Hoogeman MS, Heijmen BJ (2015). Comparison of VMAT and IMRT strategies for cervical cancer patients using automated planning. Radiother Oncol.

[18] Cao Y, Zhu X, Ju X, Liu Y, Yu C, Sun Y (2018). Optimization of dose distributions of target volumes and organs at risk during stereotactic body radiation therapy for pancreatic cancer with dose-limiting auto-shells. Radiat Oncol.

[19] Duan Y, Gan W, Wang H, Chen H, Gu H, Shao Y (2020). On the optimal number of dose-limiting shells in the SBRT auto-planning design for peripheral lung cancer. J Appl Clin Med Phys.

[20] Gay HA, Niemierko A (2007). A free program for calculating EUD-based NTCP and TCP in external beam radiotherapy. Phys Med.

[21] Niemierko A (1997). Reporting and analyzing dose distributions: a concept of equivalent uniform dose. Med Phys.

[22] Sukhikh ES, Sukhikh LG, Lushnikova PA, Tatarchenko MA, Abdelrahman AR (2020). Dosimetric and radiobiological comparison of simultaneous integrated boost and sequential boost of locally advanced cervical cancer. Phys Med.

[23] Cao T, Dai Z, Ding Z, Li W, Quan H (2019). Analysis of different evaluation indexes for prostate stereotactic body radiation therapy plans: conformity index, homogeneity index and gradient index. Precis Radiat Oncol.

[24] Tezcan Y, Koc M (2011). Gastrointestinal stromal tumor of the rectum with bone and liver metastasis: a case study. Med Oncol.

[25] Di Scioscio V, Greco L, Pallotti MC, Pantaleo MA, Maleddu A, Nannini M (2011). Three cases of bone metastases in patients with gastrointestinal stromal tumors. Rare Tumors.

[26] Gatto L, Nannini M, Saponara M, Di Scioscio V, Beltramo G, Frezza GP (2017). Radiotherapy in the management of gist: state of the art and new potential scenarios. Clin Sarcoma Res.

[27] Joensuu H, Eriksson M, Collan J, Balk MH, Leyvraz S, Montemurro M (2015). Radiotherapy for GIST progressing during or after tyrosine kinase inhibitor therapy: a prospective study. Radiother Oncol.

[28] Strander H, Turesson I, Cavallin-Stahl E (2003). A systematic overview of radiation therapy effects in soft tissue sarcomas. Acta Oncol.

[29] Schültke E, Balosso J, Breslin T, Cavaletti G, Djonov V, Esteve F (2017). Microbeam radiation therapy—grid therapy and beyond_a clinical perspective. Br J Radiol..

[30] Zhang H, Wu X, Zhang X, Chang SX, Megooni A, Donnelly ED (2020). Photon GRID radiation therapy: a physics and dosimetry white paper from the radiosurgery society (RSS) GRID/LATTICE, Microbeam and FLASH radiotherapy working group. Radiat Res.

[31] Jiang L, Li X, Zhang J, Li W, Dong F, Chen C (2020). Combined high-dose LATTICE radiation therapy and immune checkpoint blockade for advanced bulky tumors: the concept and a case report. Front Oncol.

[32] Yu W, Cai XW, Liu Q, Zhu ZF, Feng W, Zhang Q (2015). Safety of dose escalation by simultaneous integrated boosting radiation dose within the primary tumor guided by (18)FDG-PET/CT for esophageal cancer. Radiother Oncol.

[33] Wang D, Bi N, Zhang T, Zhou Z, Xiao Z, Liang J (2019). Comparison of efficacy and safety between simultaneous integrated boost intensity-modulated radiotherapy and conventional intensity-modulated radiotherapy in locally advanced non-small-cell lung cancer: a retrospective study. Radiat Oncol.

[34] Nomiya T, Akamatsu H, Harada M, Ota I, Hagiwara Y, Ichikawa M (2015). Modified simultaneous integrated boost radiotherapy for unresectable locally advanced breast cancer: preliminary results of a prospective clinical trial. Clin Breast Cancer.

[35] Nomiya T, Akamatsu H, Harada M, Ota I, Hagiwara Y, Ichikawa M (2014). Modified simultaneous integrated boost radiotherapy for an unresectable huge refractory pelvic tumor diagnosed as a rectal adenocarcinoma. World J Gastroenterol.

[36] Cilla S, Deodato F, Ianiro A, Macchia G, Picardi V, Buwenge M (2018). Partially ablative radiotherapy (PAR) for large mass tumors using simultaneous integrated boost: a dose-escalation feasibility study. J Appl Clin Med Phys.

[37] Rasmussen JH, Hakansson K, Vogelius IR, Aznar MC, Fischer BM, Friborg J (2016). Phase I trial of 18F-Fludeoxyglucose based radiation dose painting with concomitant cisplatin in head and neck cancer. Radiother Oncol.

[38] Pigorsch SU, Wilkens JJ, Kampfer S, Kehl V, Hapfelmeier A, Schlager C (2017). Do selective radiation dose escalation and tumour hypoxia status impact the loco-regional tumour control after radio-chemotherapy of head & neck tumours? The ESCALOX protocol. Radiat Oncol.

[39] Blanck O, Wang L, Baus W, Grimm J, Lacornerie T, Nilsson J (2016). Inverse treatment planning for spinal robotic radiosurgery. J Appl Clin Med Phys.

